# DNA Repair and Cancer Therapy: Targeting APE1/Ref-1 Using Dietary Agents

**DOI:** 10.1155/2012/370481

**Published:** 2012-09-10

**Authors:** Julian J. Raffoul, Ahmad R. Heydari, Gilda G. Hillman

**Affiliations:** ^1^Department of Medicine, Emory University School of Medicine, Atlanta, GA 30322, USA; ^2^Department of Nutrition and Food Science, Barbara Ann Karmanos Cancer Institute, Wayne State University, Detroit, MI 48202, USA; ^3^Department of Radiation Oncology, Barbara Ann Karmanos Cancer Institute, Wayne State University School of Medicine, Detroit, MI 48201, USA

## Abstract

Epidemiological studies have demonstrated the cancer protective effects of dietary agents and other natural compounds isolated from fruits, soybeans, and vegetables on neoplasia. Studies have also revealed the potential for these natural products to be combined with chemotherapy or radiotherapy for the more effective treatment of cancer. In this paper we discuss the potential for targeting the DNA base excision repair enzyme APE1/Ref-1 using dietary agents such as soy isoflavones, resveratrol, curcumin, and the vitamins ascorbate and **α**-tocopherol. We also discuss the potential role of soy isoflavones in sensitizing cancer cells to the effects of radiotherapy. A comprehensive review of the dual nature of APE1/Ref-1 in DNA repair and redox activation of cellular transcription factors, NF-**κ**B and HIF-1**α**, is also discussed. Further research efforts dedicated to delineating the role of APE1/Ref-1 DNA repair versus redox activity in sensitizing cancer cells to conventional treatment are warranted.

## 1. Introduction

Despite the “war on cancer” initiated by the signing of the National Cancer Act of 1971, cancer remains a major public health concern in the United States accounting for approximately 1 in 4 deaths [1]. Current conventional cancer treatment involves the use of chemotherapy or radiotherapy, either alone or in combination. The central mechanism by which chemotherapy or radiation exert their cytotoxic effects is directly related to their ability to cause DNA damage. Limitations exist, however, for these treatment options when used as single modalities for most solid tumors as cancer cells are known to be highly heterogenous and display deregulation of multiple cellular signaling pathways. In order to improve cancer treatment outcomes, new strategies must be investigated. Novel concepts include using targeted therapies and combining drugs with dietary agents for improved cancer cell death and reduced residual toxicity. 

Sensitizing cancer cells to DNA damaging agents by targeting DNA repair pathways is an emerging concept that is receiving much deserved attention [2]. Efficient DNA repair is an important mechanism by which cancer cells exert therapeutic resistance. Thus, altering the ability of a cancer cell to respond to DNA damaging agents should render a cell more susceptible to death. This concept is further supported by research demonstrating that polymorphisms in DNA repair are associated with increased risk for cancer, influence the natural history and progression of the disease, and predict response to chemotherapy and radiation [3–6]. It is therefore worth pursuing new strategies of cancer therapy that target DNA repair.

Numerous studies also support the notion that diet could influence cancer development, progression, metastasis and mortality [7]. Furthermore, these studies suggest that susceptibility to various cancers is due to environmental factors, that is, diet, rather than genetic differences. The potential for herbs and other plant-based formulations to act as antioxidants has also been increasingly recognized in the prevention and treatment of cancers [8–10]. These dietary compounds include, but are not limited to, soy isoflavones, resveratrol, lycopene, thymoquinones and their derivatives, green tea polyphenols, and curcumin. All have been recognized as cancer chemopreventive agents because of their anticarcinogenic activity, yet also exert antitumor activities through regulation of different cell signaling pathways. Therefore, the use of dietary agents to potentiate conventional cancer treatment is a promising area for investigation [11–16].

The purpose of this paper is to understand the use of dietary agents, in particular soy isoflavones, as DNA repair inhibitors. Specifically, we will discuss targeting the DNA base excision repair (BER) enzyme apurinic/apyrimidinic endonuclease 1/redox-factor-1 (APE1/Ref-1), a multifunctional protein involved in both DNA repair and redox signaling, whose expression is altered in numerous cancers including prostate, colon, ovarian, cervical, and germ cell tumors [17]. Elevated levels of APE1/Ref-1 have been linked to resistance to chemotherapy, poor prognosis, and poor survival [18–20]. Selective targeting of this DNA repair enzyme using RNA interference, antisense oligonucleotides, and dietary agents has been shown to be effective in sensitizing cancer cells to both radiation and chemotherapy *in vivo* and *in vitro* [11, 12, 18–20]. Furthermore, the use of specific small-molecule inhibitors blocking either APE1/Ref-1 repair or redox functions, but not both, is currently under investigation [21]. However, research utilizing dietary approaches that inhibit DNA repair enzymes is relatively scarce and the need for further studies will be emphasized. 

## 2. APE1/Ref-1: An Overview

Apurinic/apyrimidinic (AP) endonuclease 1 (APE1) is a multifunctional protein involved in the maintenance of genomic integrity and in the regulation of gene expression. After initial discovery in *E. coli* [22], APE1 was purified from calf thymus DNA and characterized as an endonuclease that cleaves the backbone of double-stranded DNA containing AP sites [23, 24]. APE1 homologues were subsequently identified and characterized in yeast as *APN1* [25], mice as *Apex* [26, 27], and humans as *HAP1* [28]. In addition to its major 5′-endonuclease activity, APE1 expresses minor 3′-phosphodiesterase, 3′-phosphatase, and 3′→5′ exonuclease activities [29]. APE1 is the primary enzyme responsible for recognition and incision of noncoding AP sites in DNA resulting from spontaneous, chemical, or DNA glycosylase-mediated hydrolysis of the *N*-glycosyl bond initiated by the DNA base excision repair (BER) pathway. AP sites are particularly common, arising at the rate of approximately 50,000–200,000 per cell per day under normal physiological conditions [30, 31]. If unrepaired, these sites promote cell death by serving as blocks to DNA replication [32], stalling RNA transcription [33], or promoting double-strand DNA breaks [34], thus, highlighting the potential of APE1 to serve as a target for cancer therapeutics.

**Figure 1:**
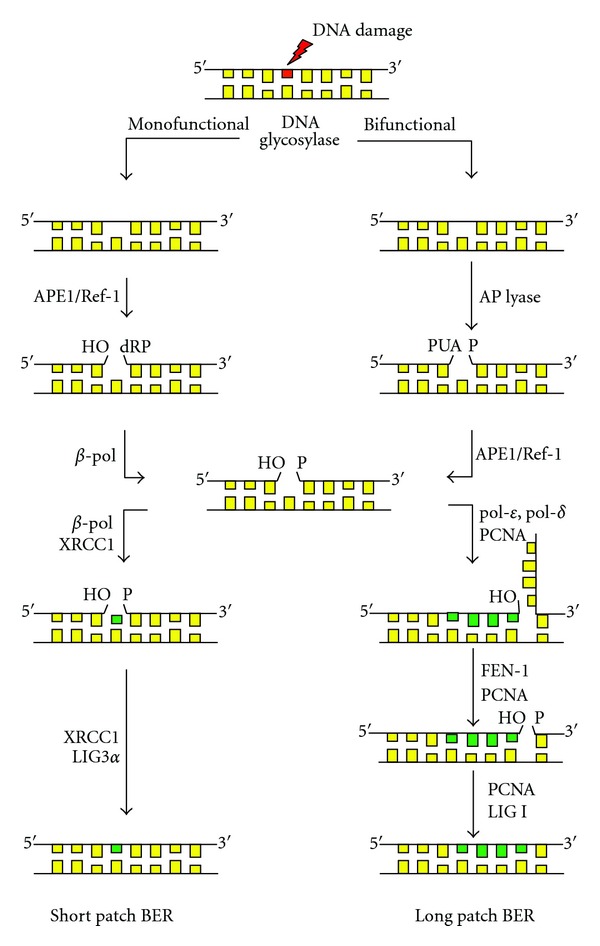
The DNA base excision repair (BER) pathway. DNA glycosylases initiate BER by recognizing and removing DNA damage forming an apurinic/apyrimidinic (AP) site. In MFG-BER, APE1/Ref-1 hydrolyzes the phosphate bond 5′ to the AP site leaving a 3′-OH group and a 5′-deoxyribose phosphate (5′-dRP) termini. DNA polymerase *β* (*β*-pol) then excises the 5′-dRP moiety generating a 5′-phosphate (5′-P). If the pathway is initiated by a bifunctional DNA glycosylase, removal of the damaged base and AP site formation is followed by AP lyase activity that hydrolyzes the 3′-bond to the AP site, resulting in a phospho-*α*,*β*-unsaturated aldehyde AP site (PUA). APE1/Ref-1 processes this site resulting in a 3′-OH group. BER then proceeds via short-patch or long-patch BER. In short-patch BER, *β*-pol inserts a single nucleotide in the AP site and LigIII*α* ligates the DNA backbone. In long-patch BER, pol *δ*/*ε* inserts 2-8 nucleotides in the AP site. The resulting DNA flap is excised by the FEN1/PCNA endonuclease complex and the DNA backbone ligated by Ligase I (LIG1).

BER is the main pathway responsible for repairing AP sites in DNA and is initiated by DNA damage recognition enzymes, that is, monofunctional or bifunctional DNA glycosylases, in addition to APE1-mediated AP site recognition (Figure 1). In monofunctional glycosylase-initiated BER (MFG-BER), a damaged or improper base is recognized and removed by enzymatic hydrolysis of the *N*-glycosyl bond resulting in the formation of an AP site. This serves as a substrate for APE1 which then incises the DNA backbone immediately 5′ to the AP site via its 5′-endonuclease activity, producing a single-strand break with a normal 3′-hydroxyl group and an abnormal 5′-deoxyribose-5-phosphate (dRP) residue [35]. DNA polymerase *β* (*β*-pol) then inserts a new base followed by the coupled excision of the abnormal 5′-dRP (Figure 1) [36]. In bifunctional glycosylase-initiated BER (BFG-BER), a damage-specific DNA glycosylase recognizes and removes the damaged base followed by incision of the DNA backbone by the associated AP lyase activity, yielding a normal 5′-terminal deoxynucleoside-5′-phosphate residue and an abnormal 3′-terminal *α*,*β*-unsaturated aldehyde residue that must be processed by APE1 3′-phosphodiesterase activity prior to repair completion (Figure 1) [35, 37]. BER may then proceed by one of two pathways: (i) short patch BER, a *β*-pol-mediated single nucleotide insertion, similar to MFG-BER, or (ii) long patch BER, that is, multiple nucleotide strand-displacement synthesis which is required to process modified (i.e., reduced, oxidized) AP sites and involves components of the DNA replication machinery [38]. Repair is completed upon the nick-sealing activity of DNA ligase complexes (Figure 1) [39].

**Figure 2:**
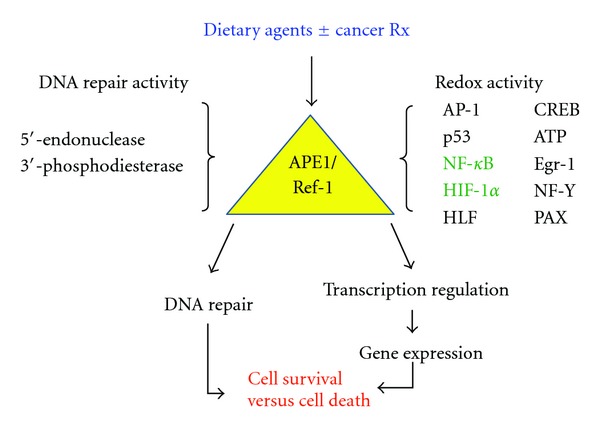
The dual functions of APE1/Ref-1. As a DNA repair protein, APE1/Ref-1 functions as the primary enzyme responsible for recognition and repair of mutagenic apurinic/apyrimidinic (AP) sites in DNA as part of the base excision repair (BER) pathway. As a redox protein, APE1/Ref-1 functions as an activator of transcription factors involved in multiple cellular processes, including AP-1, p53, NF-*κ*B, HIF-1*α*, and others. Activation involves the reduction of a cysteine residue to a sulfhydryl state. Dietary agents may target APE1/Ref-1 DNA repair or redox activities or both, causing multiple downstream effects.

Independent of its discovery as a DNA repair protein, APE1 was also characterized as Ref-1, for redox effector factor-1, the nuclear factor responsible for reducing the transcription factor AP-1 [40, 41]. Since this initial discovery, APE1/Ref-1 was characterized as a redox activator of a number of additional transcription factors known to be involved in cancer cell signaling, such as NF-*κ*B, HIF-1*α*, p53, and others (Figure 2) [17]. While the exact mechanism of the redox change has yet to be elucidated, it is known that oxidation of a cysteine residue abolishes DNA binding, whereas reduction to a sulfhydryl state promotes DNA binding [42]. The redox state of cysteine residues also influences the various properties of proteins, including protein stability, structure, and enzymatic activity [42]. The discovery of APE1/Ref-1 as a regulator of transcriptional activity could underscore the importance of its involvement in an array of physiological functions including cellular growth and differentiation, cell cycle control, apoptosis, and angiogenesis. All of which have implications for the development of cancer therapeutics. 

## 3. Phenotypic Effect of APE1/Ref-1

The importance of APE1/Ref-1 in normal cellular function is highlighted by research demonstrating the embryonic lethality of mice with homozygous deletion of the APE1 gene (Apex^−/−^), but heterozygous mice survive and are fertile (Apex^+/−^) [43–45]. Cell lines completely deficient for APE1/Ref-1 are also nonviable and further demonstrate its importance in cell survival and propagation [45]. Pursuing APE1/Ref-1 inhibition as a strategy for cancer cell therapy is justified based on the following observations. (1) APE1/Ref-1 expression and/or activity are upregulated or dysregulated in many types of cancer, including prostate, ovarian, cervical, pancreatic, colon, germ cell tumors, and rhabdomyosarcomas [17–20, 46–48]. (2) Reduction in APE1/Ref-1 using RNA interference or antisense technology *in vitro *or in studies with Apex^+/−^ mice potentiates the cytotoxicity of many laboratory and clinical DNA damaging agents including methylmethane sulfonate (MMS), H_2_O_2_, 2-nitropropane (2-NP), bleomycin, temozolomide (TMZ), melphalan, cisplatin, gemcitabine, and radiation [17–20, 46–48]. (3) Elevated expression of APE1/Ref-1 is associated with increased resistance to radiation and chemotherapy, incomplete treatment response, poor survival and prognosis, and increased level of angiogenesis [17–20, 46–48]. 

We have extensively characterized a mouse containing a heterozygous gene-targeted deletion of the APE1/Ref-1 gene (Apex^+/−^) [48, 49]. Our studies demonstrated that APE1/Ref-1 haploinsufficient (Apex^+/−^) mice show tissue-specific differences in BER capacity as characterized by an *in vitro *G : U mismatch repair assay. Others have shown that these mice display increased spontaneous mutagenesis in liver and spleen [50]. Furthermore, embryonic fibroblasts and brain cells obtained from Apex^+/−^ mice are more susceptible to oxidative stress [51].

Previous studies have indicated that downregulation of APE1/Ref-1 may promote a DNA damage-hypersensitive phenotype [51]. To determine the functional importance of decreased APE1/Ref-1 in haploinsufficient mice, we analyzed the effect of reduced APE1/Ref-1 on 2-Nitropropane-(2-NP-) induced oxidative DNA damage *in vivo* [47, 48]. 2-NP is a known hepatocarcinogen and inducer of oxidative DNA damage in the form of increased 8-hydroxydeoxyguanosine, DNA single-strand breaks, p53 levels, and *β*-pol expression and BER activity *in vivo* [47]. Previously, we have measured the presence of AP sites, single-strand breaks, and aldehydic lesions in isolated liver DNA from APE1/Ref-1 haploinsufficient mice and observed no significant difference in DNA damage accumulation as a result of reduced APE1/Ref-1 [49]. The lack of damage accumulation in untreated Apex^+/−^ mice suggested that APE1/Ref-1 haploinsufficiency in liver does not cause an accumulation of genotoxic DNA repair intermediate products under baseline conditions. In line with previous studies from our laboratory [47], we have demonstrated a significant increase in 3′-OH-containing single-strand breaks in response to oxidative stress. However the level of detectable single strand breaks (SSB's) in the liver tissue of 2-NP-treated Apex^+/−^ mice was found to be significantly lower than its wildtype counterpart while the level of aldehydic lesion was significantly higher. We suggest that the processing of oxidized bases by a bifunctional DNA glycosylase such as OGG1 (8-oxoguanine DNA glycosylase) could result in generation of aldehydic blocking lesions at 3′ end. Inability to process these 3′ blocking groups in the absence of the 3′-phosphodiesterase activity of Apex in Apex^+/−^ mice [37], could result in lower detection of endonuclease-mediated single-strand breaks in the heterozygous animal.

Reports to date have shown that APE1/Ref-1 is inducible in response to various forms of oxidative stress [49, 51–58]; however, it is currently unclear whether this response is due to APE1/Ref-1 repair activity versus redox regulatory activity, or both. Our studies in Apex^+/−^ mice indicate that APE1/Ref-1 is indeed an inducible protein, with concomitant changes in NF-*κ*B, emphasizing its role as a redox protein [48]. We have confirmed that APE1/Ref-1 is indeed an inducible protein [48]. While fold increase is the same in response to oxidative stress across the genotypes, the total accumulative level of APE1/Ref-1 protein is lower in the liver of Apex^+/−^ mice; that is, even though the intact allele is induced in response to 2-NP, it does not compensate for the lost allele. In line with these findings, the Apex^+/+^ mice showed a significant increase in APE1/Ref-1 redox activation of NF-*κ*B when exposed to 2-NP. Thus, the ultimate level of NF-*κ*B activation in response to oxidative stress was significantly attenuated in the heterozygous (Apex^+/−^) animals.

It is well established that NF-*κ*B is a mediator of inflammatory responses, promoting cell proliferation and survival by inhibiting cell cycle arrest and apoptosis. Thus, reduced activation of NF-*κ*B (and possibly other APE1/Ref-1 redox-dependent transcription factors) in response to oxidative stress in Apex^+/−^ mice may prove detrimental owing to alterations in the signaling pathways necessary to differentiate between DNA repair and cell survival versus apoptosis. However, although the fold increase in response to oxidative stress is the same compared to wild-type (Apex^+/+^) mice, the total cumulative level of APE1/Ref-1 protein is lower. Hence, the intact allele in wild-type mice does not compensate for the lost allele in Apex^+/−^ mice. Thus, reduced activation of NF-*κ*B (and possibly other APE1/Ref-1 redox-dependent transcription factors) in response to oxidative stress in Apex^+/−^ mice may prove detrimental owing to alterations in the signaling pathways necessary to differentiate between DNA repair and cell survival versus apoptosis.

When examining the effect of reduced APE1/Ref-1 on DNA damage accumulation, Apex^+/−^ mice expressed a BER phenotype that is more susceptible to accumulation of DNA damage in response to oxidative stress as a result of reduced APE1/Ref-1 3′-phosphodiesterase activity [48]. Reduced APE1/Ref-1 in haploinsufficient mice also resulted in a differential impact on BER, depending upon the initiating glycosylase [48]. Oxidative stress resulted in increased MFG-BER initiated by uracil DNA glycosylase (UDG), but a significant decline in the repair or oxidized bases (8-OHdG) initiated by OGG1 (8-oxoguanine DNA glycosylase) in BFG-BER (Figure 1) [48]. The failed upregulation of BFG-BER and accumulation of repair intermediates in Apex^+/−^ mice exposed to oxidative stress coincided with increased cell death as indicated by increased expression of apoptotic markers such as GADD45g, p53, and caspase-3 activity [48].

Taken together, these results indicate that when APE1/Ref-1 is compromised, cells become more susceptible to oxidative stress primarily as a result of reduced APE1/Ref-1 redox activity and 3′-phosphodiesterase repair activity, thus impacting cell survival and pushing cells towards apoptosis. These findings have great clinical relevance for cancer as development of therapeutics targeting APE1/Ref-1 DNA repair or redox activities, or both, could potentiate current cancer cell treatment strategies. 

Interestingly, dietary agents such as soy isoflavones have been shown to interfere with APE1/Ref-1 repair and redox activity resulting in potentiation of radiotherapy for cancer cells [11, 12, 59, 60].

## 4. Dietary Modulation of APE1/Ref-1

Epidemiological studies indicate an inverse association between cancer risk and consumption of a diet rich in fruits and vegetables [7]. The cancer-inhibitory potential of nutrient and nonnutrient components, that is, phytochemicals, from plants has been confirmed in animal models [7]. Dietary sources of phytochemicals include whole grain cereal foods, seeds, soybean products (mainly isoflavones), berries or grapes (resveratrol), and nuts (mainly lignans). Emphasizing a healthy diet is relevant for cancer prevention. In addition, the use of diet as a safe and healthy supplement to conventional cancer therapy has also gained significant interest in the scientific community. Nontoxic “natural products” found in the diet have been shown to be effective in combination with conventional agents for the treatment of cancer [11–16] and such strategies are worth exploring. Here, we will review studies using dietary agents (soy isoflavones, curcumin, and resveratrol), including antioxidants (selenium, ascorbate, and *α*-tocopherol), and discuss their effects on APE1/Ref-1.

### 4.1. Soy Isoflavones

Soy isoflavones, which include genistein, daidzein, and glycitein, are plant estrogens with potent anti-oxidant and anti-inflammatory properties. An inverse association between consumption of soy isoflavones and cancer incidence has been widely documented [11–16]. We and others have shown that soy isoflavones enhance the efficacy of chemotherapy and radiation therapy of multiple cancers models *in vitro* and *in vivo* [59, 60] and in early clinical trials [61]. In addition to their use as potent adjuvant therapies, soy isoflavones could also potentially protect normal tissues from treatment-induced toxicity [11, 12, 61] and have generated much interest in the clinical research community [16].

Soy isoflavones (or puregenistein)inhibitedAPE1/Ref-1expression in prostate cancer cells in a time- and dose-dependent manner[59]. The nuclear expression ofAPE1/Ref-1was increased by radiation, probably representing an early event in the cellresponse to radiationbecause of its role in BER [59]. Pretreatment of prostate cancer cells with soy isoflavones inhibited both the increased expression and thenuclear localizationofAPE1/Ref-1induced by radiation [59]. These data were reproduced in A549nonsmall-cell lung cancercells, demonstrating that soy isoflavones caused a decrease inAPE1/Ref-1expression and inhibited upregulation ofAPE1/Ref-1expression induced by radiation [60]. It is conceivable that inhibition of APE1/Ref-1 levels by soy isoflavones could render the cancer cells more radiosensitive. Some attempts have been made to correlate APE1/Ref-1 levels of expression with tumor sensitivity to radiation therapy as increased APE1/Ref-1 expression promoted tumor resistance to ionizing radiation [62]. Conversely, decreased APE1/Ref-1 levels in RNAi-treated human osteogenic sarcoma cells led to enhanced cell sensitization to the DNA damaging agents including ionizing radiation [63]. 

#### 4.1.1. Effect of Soy on APE1/Ref-1 DNA Repair Activity

To investigate further the role of APE1/Ref-1 function in the mechanism of interaction between soy isoflavones and radiation, the formation and repair of DNA double-strand breaks (DSBs) induced by radiation were studied. Ionizing radiation causes rapid phosphorylation of the nucleosomal histone protein H2AX at Ser 139 (*γ*-H2AX), occurring at sites of DNA DSBs, which can be visualized as fluorescent foci by immunostaining [64, 65]. Formation of *γ*-H2AX foci occurs within minutes after production of DSBs by ionizing radiation, and the loss of *γ*-H2AX foci after several hours can be attributed to DNA repair enzymes [64, 65]. In A549 nonsmall cell lung cancer cells, a large number of *γ*-H2AX foci occurred by 1 h after 3 Gy radiation, but drastically decreased at 24 h after radiation, suggesting that A549 cells activated DNA repair mechanisms. Interestingly, we found that soy isoflavones also cause DSBs [60]. However, in contrast to radiation, the number of *γ*-H2AX foci increased and persisted over time in soy isoflavones pretreated cells, probably interfering with DNA repair mechanisms [60]. Importantly, the combination of soy isoflavones and radiation caused an increase in frequency and intensity of *γ*-H2AX foci, which were maintained at 24 h, indicating both increased DNA damage and inhibition of repair [60]. Our novel findings on induction and kinetics of DSBs formation by soy isoflavones suggest that soy isoflavones disrupt DNA repair processes and potentially sensitizes nonsmall cell lung cancer cells to the cytotoxic effect of radiation [60]. Furthermore, these data are also consistent with the inhibition of the radiation-induced upregulation of the DNA repair enzyme APE1/Ref-1 by soy isoflavones in A549 cells, which could contribute to alterations in DNA repair mechanisms [60]. In contrast, cells treated with radiation alone showed a significant increase in APE1/Ref-1 within 5 h after radiation, which could be associated with the loss of *γ*-H2AX foci. 

To determine if the soy-mediated decrease inAPE1/Ref-1expression is involved in the mechanism of soy inhibition ofDNA repair, two differentAPE1/Ref-1inhibitors, E3330 andmethoxyamine, were tested [60]. E3330, a novel quinine derivative shown to inhibit the redox activity of APE1/Ref-1 [17–21], did not alter the repair of radiation-induced DSBs over time. However, methoxyamine, an alkoxyamine derivative and indirect inhibitor of APE1/Ref-1 endonuclease activity [17–21], partially blocked the decrease in radiation-induced DSBs. These data indicate partial mitigation of radiation-induced BER bymethoxyamine, akin to the effect of soy when it is combined with radiation.Methoxyaminealso increasedcell killingmediated by soy isoflavones as well as that by soy combined with radiation, suggesting that additionalDNA repairinhibition ofAPE1/Ref-1results in furthercell killing[60]. These findings suggest that inhibition of APE1/Ref-1DNA repairactivity by soy isoflavones is involved in the mechanism by which soy isoflavones potentiate radiation-induced cancer cell killing.

#### 4.1.2. Effect of Soy on APE1/Ref-1 Redox Activity

In numerous studies, NF-*κ*B was shown to be an important molecular target of soy isoflavones in cancer cells [13–16]. The inhibition of NF-*κ*B DNA binding activity by soy isoflavones alone or combined with radiation correlated with the down-regulation of APE1/Ref-1 expression [59]. Overexpression of APE1/Ref-1, obtained by cDNA transfection of PC-3 cells, caused a concomitant increase in NF-*κ*B DNA binding activity. Moreover, soy isoflavones treatment of APE1/Ref-1 overexpressing PC-3 cells significantly inhibited APE1/Ref-1 expression with a corresponding decrease in the NF-*κ*B DNA binding activity [59]. Thus, in addition to alteration of the DNA repair activity of APE1/Ref-1, soy isoflavones also affected the redox activation function of APE1/Ref-1 (Figure 2). These findings further confirm that soy isoflavones disrupt molecular cross-talks between APE1/Ref-1 and NF-*κ*B which are two critical molecules essential for cell survival pathways. 

Another critical signaling pathway upregulated by radiation-induced oxidative stress is the transcription factor hypoxia-inducible factor (HIF-1*α*), which is induced by hypoxia. HIF-1*α* is responsible for the activation of more than 60 downstream target genes involved in angiogenesis, tumor growth, and invasion [13–16]. Interestingly, APE1/Ref-1 is also responsible for redox-activation of HIF-1*α* (Figure 2). In the hypoxic response, cellular levels of HIF-1*α* and APE1/Ref-1 redox stabilization of the HIF-1*α* protein are critical for its nuclear translocation and DNA binding and transcriptional activity [61]. Studies on cellular localization of HIF-1*α* demonstrated that soy isoflavones inhibited nuclear translocation of HIF-1*α* protein, a process which is upregulated by radiation but suppressed by pretreatment with soy isoflavones [66]. Therefore, APE1/Ref-1 downregulation by soy isoflavones could play a central and pivotal role in radiosensitization of prostate cancer cells by affecting HIF-1*α* pathway. Radiation induced HIF-1*α* expression and DNA-binding activity *in vitro* but both were abrogated by pretreatment of PC-3, C4-2B, and A549 cells with soy isoflavones in [60, 66]. Therefore, soy isoflavone-mediated inhibition of HIF-1*α* activation by oxidative stress could render cancer cells more radiosensitive.

These findings demonstrate the molecular cross-talk between APE1/Ref-1, NF-*κ*B, and HIF-1*α* and indicate a critical role for APE1/Ref-1 in the mechanism of interaction between soy isoflavones and radiation that results in the inhibition of NF-*κ*B, and HIF-1*α* transcription of genes essential for tumor cell survival, tumor growth, and angiogenesis (Figure 2). Our studies confirm that soy isoflavones exert pleiotropic molecular effects in cancer cells, which result in the regulation of multiple signal transduction pathways involved in tumor cell growth and proliferation. 

### 4.2. Resveratrol

Resveratrol (3,5,4′-trihydroxy-*trans*-stilbene), is a naturally occurring polyhydroxylated stilbene that is widely present in grapes, red wine, mulberries, and other edible plants. Resveratrol prevented the development of carcinogen-induced skin cancer in mice and was effective in all stages of carcinogenesis [67]. Resveratrol has also been shown to be effective in the prevention of DMBA-induced mammary carcinogenesis [68]. Resveratrol induced prostate cancer cell apoptosis in multiple cell lines and suppressed the progression of prostate cancer in TRAMP mice [69–71]. Resveratrol was also effective against tumors of the liver, pancreas, gastrointestinal tract, lung, and soft tissues [72–76]. It has also been shown to enhance the therapeutic effects of 5-FU in a murine model of liver cancer [77]. Phase I studies with resveratrol have been promising and demonstrated the clinical safety of oral resveratrol up to 5 g per day [78]. 

Although studies have shown that resveratrol exerts protective effects against experimentally induced carcinogenesis, the molecular mechanism(s) by which this occurs is largely unknown. It is believed to act as an antioxidant. In studies using human melanoma cells, resveratrol was shown to inhibit, in a dose-dependent manner, the APE1/Ref-1-mediated DNA-binding of AP-1. Resveratrol was also shown to inhibit APE1/Ref-1 endonuclease activity and render melanoma cells more sensitive to treatment with the alkylating agent dacarbazine [79]. These findings suggest a role for APE1/Ref-1 redox and repair activity in the mechanism of action of resveratrol and the need for expanding further on these studies.

### 4.3. Curcumin

Curcumin, isolated from the plant root of *Curcuma longa*, is the major yellow pigment in turmeric, a widely used spice, and well-known medicinal agent in Southeast Asia. Curcumin has been shown to exhibit antitumor effects in multiple cancer cell lines and animal models [80] and to enhance the efficacy of chemotherapeutic drugs such as 5-FU, gemcitabine, and the vinka alkaloid vinorelbine [81–83]. Curcumin also has synergistic activity with other dietary agents such as genistein and green tea [84, 85]. A phase I clinical trial showed that curcumin is safe up to 8 grams per day [86]. 

The molecular mechanism of action of curcumin has been shown to involve the interruption of cancer initiation or suppression of tumor promotion and progression [87, 88]. Several studies have demonstrated the inhibitory effects of curcumin on colon carcinogenesis [89, 90], chemically-induced skin cancer [91], and DMBA-induced oral cancer [92]. Curcumin also inhibits the growth of different types of cancer cells *in vitro* and in xenograft models by inducing cell cycle arrest and apoptosis [93, 94]. Interestingly, a recent study evaluating the use of curcumin in protecting and treating carbon tetrachloride-induced liver fibrogenesis in rats showed that APE1/Ref-1 levels correlated with reduced markers of liver damage [95]. The mechanism of both liver fibrogenesis and carcinogenesis involves the cellular response to oxidative stress. Therefore, further study examining the effects of curcumin on APE1/Ref-1 expression and activity in cancer cells lines or tumor models is warranted, as well as to determine a synergistic effect with traditional antitumor agents.

### 4.4. Antioxidants

#### 4.4.1. Selenium

Selenium is found in plentiful amounts in dairy, eggs, fish, meat, grains, and nuts. Selenium in the form of selenocysteine is a major constituent of many antioxidants known as selenoproteins. The cancer preventive effect of selenium is believed to occur by reducing the formation of oxidative DNA damage and increasing DNA repair. The active species of selenium include its methylated metabolite selenomethionine (SeMet). 

Selenium in the form of SeMet was reported to promote BER activity by p53 activation in normal human fibroblasts *in vitro* [96]. Selenium-induced p53 activation promotes BER activity by reducing specific cysteine residues in p53. A dominant-negative APE1/Ref-1 redox mutant blocks reductive activation of p53 by selenium. Selenium was also shown to stimulate the activity of thioredoxin reductase (TRX), a selenoprotein [97]. These data suggest that selenium reduces p53 through interactions involving TR, which reduces TRX and APE1/Ref-1, as well as redox interactions between APE1/Ref-1 and p53. Selenium has also been show to inhibit DNA binding of transcription factors AP-1, NF-*κ*B, and the BER DNA glycosylase FPG [97]. These data imply that selenium may reduce cancer incidence through modulation of DNA repair, cellular redox status, and transcriptional responses to oxidative stress. It also suggests that the redox function of APE1/Ref-1 is a major component of this interaction.

#### 4.4.2. Ascorbate and Alpha-Tocopherol

DNA damage is a well known mechanism of carcinogenesis and both endogenous and exogenous sources of DNA damage, including oxidative DNA damage, have been extensively characterized [98]. Many natural compounds found in the diet exert anti-oxidant effects and have been under extensive investigation for their cancer chemopreventive potential [99]. These agents are believed to act by reducing the oxidative burden in cells as well as to promote increased DNA repair. APE1/Ref-1 is a key enzyme in the repair of oxidative DNA damage and studies have shown that mice heterozygous for the APE1/Ref-1 gene are abnormally sensitive to increased oxidative stress and exhibit increased biomarkers of oxidative stress and reduced survival [100].

 Anti-oxidants such as ascorbate (vitamin C) and *α*-tocopherol (vitamin E) are found in citrus fruits, broccoli, and tomatoes and have been reported to initiate physiological responses that lower cancer risk by scavenging free radicals or reacting with their byproducts [99]. Supplementation of APE1/Ref-1 heterozygous mice with ascorbate and *α*-tocopherol restored the biomarkers of oxidative stress to normal and improved longevity in these mice [43]. These results were consistent with the hypothesis that humans with an APE1/Ref-1 deficiency are more susceptible to cancer through promotion of a DNA damage phenotype and that a diet rich in fruits and vegetables is protective, when DNA repair is compromised.

## 5. Conclusion and Future Directions

The use of nutrition intervention as an adjuvant to conventional cancer therapy has great therapeutic potential. Several studies have been referenced demonstrating the effect of natural dietary agents on the inhibition of cancer cell growth and their role in the prevention of neoplasia. These effects involve the modulation of multiple DNA repair genes, including but not limited to APE1/Ref-1, and genes involved in cell cycle progression, apoptosis, and the regulation of tumor  cell invasion and metastasis [2]. 

In this paper, we discussed the use of dietary agents in targeting APE1/Ref-1 in order to enhance cancer therapy and prevention. APE1/Ref-1 is constitutively activated in cancer cells and upregulated further inresponse to certain chemotherapeutics and radiation damage, but is inhibited by dietary agents, such as soy isoflavones, leading to increased cell killing and tumor growth inhibition. We suggest that APE1/Ref-1, a protein involved in both DNA repairand redox activation of transcription factors such as NF-*κ*B and HIF-1*α*, could play a critical role in the mechanism of interaction between dietary agents and radiation or chemotherapeutic agents. Dietary targeting of APE1/Ref-1  inhibited radiation-induced activation of bothits DNA repair and redox activities, thereby blocking the transcription of genes essential for tumor  cell survival, growth, and  angiogenesis [11–16, 59, 60]. The dual nature of APE1/Ref-1 could promote repair of damage on promoter sites, possibly incurred in hypoxic tumor microenvironments, while simultaneously reducing  transcription factors, thus ensuring proper transcription factor complex formation and gene expression (Figure 2). Therefore, simultaneous downregulation of transcription factors in cancer cells by inhibition of APE1/Ref-1 with dietary agents could decrease both cell survivaland enhance tumor radio- and chemo-sensitivity.

Sensitization of tumor cells to radiation or chemotherapy by dietary agents could also effectively combat cancer by reducing the tumor burden while simultaneously relieving normal tissue toxicity, thus reducing the adverse effects of therapy. This hypothesis was supported by our clinical trial inprostate cancer patients showing that patients receiving soy isoflavones during and after radiation therapy showed better PSA level reduction and decreased incidence of urinary, gastrointestinal, and erectile dysfunction compared to those patients receiving placebo [100]. Our preclinical *in vitro* and *in vivo* studies suggest that the anticancer properties of soy isoflavones could be better exploited if these natural compounds are used as a complementary approach to conventional radiotherapy [59, 60]. 

The *in vitro* molecular effects of dietary agents on APE1/Ref-1 expression and activity need to be further studied *in vivo*. Our findings studying the effect of soy isoflavones on APE1/Ref-1, NF-*κ*B, andHIF-1*α* demonstrated that these molecules truly represent potential biological targets for cancer therapies. Future research directions should include elucidation of the molecular mechanisms of the differential effects of dietary agents acting as adjuvants for cancer cell therapy and as antioxidants for normal tissues. Further studies are warranted to determine the role of soy isoflavones, resveratrol, curcumin and the use of dietary antioxidants as chemo- and radioenhancers for tumors and radioprotectors for normal tissues in preclinical tumor models. This is particularly needed for critical cancer sites includinglung, head andneck, andbrain, sites in which treatment-induced injury to normal surrounding tissues result in serious early and late effects. Combination therapies for advanced cancers, including radiotherapy and chemotherapy, could benefit from a complementary and safe approach using dietary agents to mitigate the adverse effects of these therapies on normal tissues and are under active clinical investigation [10]. Elucidation of the mechanisms of interaction between dietary agents and conventional cancer treatments will have a strong impact on understanding the basic science of cancer chemoprevention and will justify the continued clinical use of dietary agents as an adjuvant to standard cancer treatment.
